# The impact of COVID-19 on the interpretation of psycho-oncological support trial results: a quasi-experimental approach using the data from the new form of care “Integrated cross-sectoral psycho-oncology (nFC-isPO)”

**DOI:** 10.1186/s12913-023-09544-y

**Published:** 2023-05-30

**Authors:** Anna Hagemeier, Anne Adams, Theresia Krieger, Sandra Salm, Natalia Cecon-Stabel, Antje Dresen, Martin Hellmich

**Affiliations:** 1 Institute of Medical Statistics and Computational Biology (IMSB), Faculty of Medicine and University Hospital Cologne, University of Cologne, Cologne, Germany; 2 Medicine Faculty, Human Sciences Faculty, Institute for Medical Sociology, Health Services Research, and Rehabilitation Science (IMVR), University of Cologne, University Hospital Cologne, Cologne, Germany; 3 Goethe University Frankfurt, Institute of General Practice, Frankfurt, Germany

**Keywords:** Anxiety, cancer, COVID-19, Depression, HADS, Mental health, Psycho-oncology, Pandemic, Regression discontinuity design, Stratification

## Abstract

**Objective:**

In addition to the common difficulties of ongoing trials, the COVID-19 pandemic posed several challenges to scientists worldwide and created an additional burden for vulnerable patient groups. In the nFC-isPO of individualised treatment for anxiety and depression in newly diagnosed patients with cancer caregivers (e.g. psycho-oncologists) reported elevated HADS scores in newly enrolled patients after the outbreak of the COVID-19 pandemic. Accordingly, the question arises whether the pandemic affected HADS scores. Therefore, stratified analyses by the time of enrolment (T1) were performed for patients with 12 months of care (T3).

**Methods:**

Patients with 12 months of care (N = 1,140) were analysed. A comparison within the regression discontinuity design according to the time points at which patients completed the baseline (T1) HADS questionnaire was conducted to examine differences between patients recruited before Q2/2020 (pre-pandemic) and after the coronavirus outbreak. Furthermore, mean HADS scores at T1 and T3 for all quarters during the study were compared.

**Results:**

Mean T1 and T3 HADS scores of patients with cancer during the pandemic are only slightly higher than those of the pre-pandemic group. No significant treatment effect was observed in either the pre-pandemic (p = 0.5495, Late = 1.7711) or the post-pandemic group (p = 0.9098, LATE=-0.2933). In contrast, the average local treatment effect in the post-pandemic group suggests a minimal decrease in HADS score in the predefined range and thus a positive treatment effect for isPO. Comparison of mean HADS scores at T1 and T3 did not show a large increase by pandemic-related timepoints, however, a decrease of approximately 2–3 points over each quarter at 12 months compared to baseline is observed.

**Conclusion:**

The existing nFC-isPO care is resilient to crisis and may counteract external influences such as the Corona pandemic. Accordingly, the pandemic had little influence on the fears of patients with cancer in the nFC-isPO. This emphasises that psycho-oncology is vital for the reduction of stress, anxiety and depression in patients with cancer.

**Trial Registration:**

The study was registered in the German Clinical Trials Registry on 30 October 2018 under the ID “DRKS00015326”.

## Introduction

COVID-19 has taken a toll on people and health systems around the world, and many studies have examined this novel disease and its effects [1]. In addition to the general impact and the impact on the health system, COVID-19 also affected ongoing trials, e.g. in form of changes in the shape of care supply. These challenges were thus in addition to the usual challenges such as slow recruitment or managing multi-site. However, these problems with trials did not only affect providers, but especially vulnerable patient groups who had a higher risk of infection due to pre-existing conditions and a correspondingly weakened immune system [2–4]. One of these vulnerable groups was patients with cancer, especially those who received their first diagnosis during the pandemic. A cancer diagnosis is already a stressful and drastic event and often leads to stress, depression and especially anxiety. When patients receive such a diagnosis in an exceptional situation such as the COVID-19 pandemic, psychological stress is compounded in many cases [5, 6]. For this reason, holistic psycho-oncological treatment, i.e. in addition to treating the cause of the cancer, the inclusion of mental health and its treatment is an important component [7].

The project and the new form of care (nFC) “Integrated cross-sectoral psycho-oncology” (isPO) started in 2017 with precisely this intention, namely holistic psycho-oncological care and above all with the aim of creating structured needs-oriented psycho-oncological care in Germany, and was then challenged by the consequences of the pandemic during the project period [8, 9]. isPO was conducted at four sites in North-Rhein Westphalia using a quasi-experimental study design, the regression discontinuity design (RDD) in combination with the Hospital Anxiety and Depression Scale (HADS) to assess individual psycho-oncological needs [10]. The new form of care aims to successfully reduce anxiety and depression within the first year after cancer diagnosis in patients with cancer through psychological, psychotherapeutical and psychosocial measures. Given the study design and psychological treatment, the aim is to determine whether HADS scores decrease in the intervention group. During the study, after the COVID-19 pandemic onset (March 2020) [11], the four isPO care networks provided feedback that higher HADS values were observed in many patients, and the potential for anxiety and depression increased [12]. Therefore, the question arose as to how the influence of the pandemic can be taken into account in the evaluation of the study. This should be considered with regard to effectiveness in terms of an increase in HADS scores, but also with regard to necessary adjustments in care, such as switching from face-to-face care to care via telephone and video telephony. The aim is to discuss whether the higher HADS values observed by caregivers are reflected in the study results by performing stratified analyses (descriptive and using RDD) according to the time of enrolment in the study programme (T1).

## Materials and methods

### General study information and study design

In isPO, newly diagnosed patients with cancer were offered psycho-oncological care at different levels of care according to their individual needs which are assessed using the HADS. Patients were requested to complete the HADS questionnaire at three different time points (Baseline (T1), after 4 months of care (T2) and after 12 months of care (T3)). T1-HADS scores are used for assignment to the different levels of care and thus for classification into control and intervention groups. This is done as a quasi-randomization using the RDD, which uses a threshold criterion to divide patients into control and treatment groups and measure treatment effect by comparing the intercepts of regression equations above and below a predefined threshold and within a bandwidth [10, 13]. In isPO patients with baseline HADS values ≤ 14 were allocated to the control group (psychosocial intervention). Patients with higher values (≥ 15) received psycho-oncological, psychotherapeutical treatment (intervention group). T2 and T3 (primary endpoint) are used to compare the allocated patients around the threshold and measure their psychological needs after receiving the individual treatment. Therefore, patients with less distress at the first time point were compared to those with higher distress. This procedure aims to record and review the effectiveness of psycho-oncological psychotherapeutical care [8, 9].

### Data collection and analysis population

N = 1,757 newly diagnosed patients with cancer were recruited in the period 01/2019-03/2021. Data were collected at four isPO care networks (Cologne, Troisdorf, Mönchengladbach and Neuss) in North Rhine-Westphalia (NRW) and recorded in a computer-assisted assistance system (CAPSYS^2020^) developed for the isPO project. For proper analysis of stratified data and primary end point at T3, only cases followed for 12 months were considered (N = 1,140). The main analysis (RDD) compares the HADS scores of N = 203 patients within the two strata (T1 pre-pandemic (N = 96) vs. T1 post-pandemic (N = 107)) within the pre defined bandwidth of 13–16 points at baseline. All patients provided written informed consent.

**Fig. 1:**
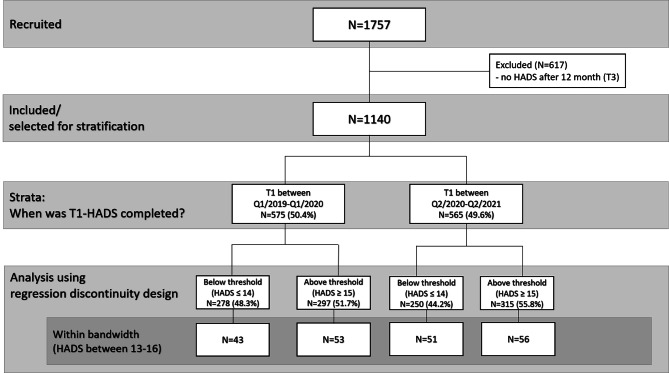
Flowchart of study and analysis population

A detailed overview of the study and analysis population is shown in Fig. 1.

### Instruments

The main instrument of the isPO study was the Hospital Anxiety and Depression Scale (HADS). The HADS consists of 14 items, 7 of which are assigned to the depression subscale and 7 to the anxiety subscale with a score from 0 to 3 for each response. The added values of the individual items result in the subscales, whereby values between 0 and 7 are to be interpreted as inconspicuous, values from 8 to 10 as suspicious and values > 10 as conspicuous. The total score ranges from 0 to 42. Based on the interpretation of the subscales, 14.5 was chosen as the threshold for group assignment for the regression discontinuity design within the nFC-isPO [14, 15].

### Statistics

Simple stratification by time intervals was used to account for the pandemic influence. Therefore, an additional categorical variable in the database was built. The variable considers at what point in the study a patient completed the HADS questionnaire at timepoint T1 (baseline). This variable was then used to stratify patients into a pre- and post-pandemic groups (T1 in Q1/19-Q1/20 vs. T1 in Q2/20-Q2/21), to identify differences between patients´ anxiety and depression levels over time and to explore possible differences during the chronological trend.

First, descriptive analyses were conducted to provide an overview of the study population. Therefore, categorical variables are reported as absolute and relative frequencies in percentage. For continuous variables mean with standard deviation and median with interquartile range are given. Next, RDD analysis were conducted in the two strata to measure the effect of the psycho-oncological treatment. An RDD analysis is based on simple linear regression in which two regression equations are compared below and above a threshold [10]. The difference between the two regression lines or the difference between the two axis intercepts at the pre defined threshold then gives the treatment effect. This is also called the local average treatment effect (LATE) and is graphically represented as a discontinuity (jump) of the regression lines at the threshold [10, 13].

In addition, mean baseline HADS scores were compared with HADS scores after 12 months of care to show changes over time. All analyses were performed using IBM SPSS Version 28 with available R extensions, e.g. regression discontinuity analysis and figures were generated with R Version 4.1.2. [16] Results were considered statistically significant for p ≤ 0.05 with α = 0.05 as the significance level.

## Results

### Characteristics of the patients

A total of 1,140 patients completed the HADS questionnaire after 12 months. Of these, 575 completed the HADS for the first time between Q1/19-Q1/20, before the pandemic, and 565 between Q2/20-Q2/21, after the pandemic outbreak. The gender ratio was about two-thirds women to one-third men with a median age of about 58 years (IQR=[50–66], mean = 56.8, SD = 13.1). The most common diagnoses were breast cancer (30%) and malignant neoplasms of the digestive organs (13.3%). An overview of all sociodemographic and clinical patient characteristics is given in Table 1.

**Table 1:** Sociodemographic and clinical patient characteristics by T1-time intervals

	Q1/19-Q1/20 (N = 575)	Q2/20-Q2/21 (N = 565)	Total (N = 1,140)
**Gender**			
Female	378 (65.7%)	362 (64.1%)	740 (64.9%)
Male	197 (34.3%)	203 (35.9%)	400 (35.1%)
**Age**			
Mean ± SD	56.7 ± 12.9	57.0 ± 13.3	56.8 ± 13.1
Median[Q1;Q3]	58 [50; 66]	59 [49;65]	58 [50;66]
**Care level**			
1	54 (9.4%)	50 (8.8%)	104 (9.1%)
2	224 (39.0%)	201 (35.6%)	425 (37.3%)
3a	94 (16.3%)	101 (17.9%)	195 (17.1%)
3b	203 (35.3%)	213 (37.7%)	416 (36.5%)
**Relationship status**			
No relationship	117 (21.2%)	134 (24.7%)	251 (22.9%)
In relationship, shared household	390 (70.7%)	377 (69.4%)	767 (70.0%)
In relationship, separate households	45 (8.2%)	32 (5.9%)	77 (7.0%)
**ISCED**			
Primary education	9 (1.6%)	9 (1.6%)	18 (1.6%)
Lower secondary education	38 (6.8%)	28 (5.1%)	66 (6.0%)
Upper secondary education	389 (69.7%)	385 (70.3%)	774 (70.0%)
Bachelor or equivalent	33 (5.9%)	37 (6.8%)	70 (6.3%)
Master or equivalent	80 (14.3%)	84 (15.3%)	164 (14.8%)
Doctoral or equivalent	9 (1.6%)	5 (0.9%)	14 (1.3%)
**Servere disability**			
No	393 (69.8%)	386 (69.7%)	779 (69.7%)
Yes	170 (30.2%)	168 (30.3%)	338 (30.3%)
**Chronic illness**			
No	328 (58.3%)	327 (59.0%)	655 (58.6%)
Yes	235 (41.7%)	227 (41.7%)	462 (41.4%)
**ICD10-entities**			
C15-C26	74 (13.1%)	75 (13.6%)	149 (13.3%)
C30-C39	49 (8.7%)	49 (8.9%)	98 (8.8%)
C43-C44	41 (7.2%)	29 (5.3%)	70 (6.3%)
C50	177 (31.3%)	158 (28.6%)	335 (30.0%)
C51-C58	26 (4.6%)	42 (7.6%)	68 (6.1%)
C81-C96	65 (11.5%)	54 (9.8%)	119 (10.6%)
Other (each < 5%)	134 (%)	145 (%)	279 (24.5%)
**Death**			
Yes	11 (1.9%)	1 (0.2%)	12 (1.1%)
No	564 (98.1%)	564 (99.8%)	1,128 (98.9%)

### Modification of HADS

The mean and median baseline (T1) HADS scores of the anxiety subscale are minimally higher in the group during the pandemic (9.0 ± 4.4 and 9 [6;12]) than in the group before the pandemic (8.8 ± 4.6 and 9 [5;12]). The same applies to the depression subscale (7.1 ± 4.6 and 6 [4;10] vs. 6.5 ± 4.5 and 6 [3;9]). However, for both groups (before and during the pandemic), it is visible that the T3 HADS decreases to a similar extent, by about 2 points each for the anxiety subscale and by about 1 point each for the depression subscale (see Table 2).

**Table 2:** Summary of the subscales and total HADS scores at baseline (T1) and at the primary endpoint after 12 months of care (T3) for the pre- and post-pandemic groups

	Q1/19-Q1/20 (N = 575)	Q2/20-Q2/21 (N = 565)	Total (N = 1,140)
**HADS anxiety T1**			
Mean ± SD	8.8 ± 4.6	9.0 ± 4.4	8.9 ± 4.5
Median [Q1;Q3]	9 [5;12]	9 [6;12]	9 [6;12]
**HADS anxiety T3**			
Mean ± SD	7.2 ± 4.3	7.6 ± 4.3	7.4 ± 4.3
Median [Q1;Q3]	7 [4;10]	7 [4;11]	7 [4;10]
**HADS depression T1**			
Mean ± SD	6.5 ± 4.5	7.1 ± 4.6	6.8 ± 4.6
Median [Q1;Q3]	6 [3;9]	6 [4;10]	6 [3;10]
**HADS depression T3**			
Mean ± SD	5.5 ± 4.4	6.2 ± 4.8	5.8 ± 4.6
Median [Q1;Q3]	5 [2;8]	5 [2;10]	5 [2;9]
**HADS total T1**			
Mean ± SD	15.2 ± 8.5	16.0 ± 8.4	15.6 ± 8.4
Median [Q1;Q3]	15 [8;21]	16 [10;22]	15 [9;21]
**HADS total T3**			
Mean ± SD	12.7 ± 8.2	13.8 ± 8.5	13.2 ± 8.4
Median [Q1;Q3]	12 [6;18]	13 [7;20]	12 [6;19]
**Group by HADS-Cut-off**			
HADS < = 14	278 (48.3%)	250 (44.2%)	528 (46.3%)
HADS > = 15	297 (51.7%)	315 (55.8%)	612 (53.7%)

* HADS: Hospital anxiety and depression scale

### Primary outcome pre- vs. post-pandemic

**Fig. 2:**
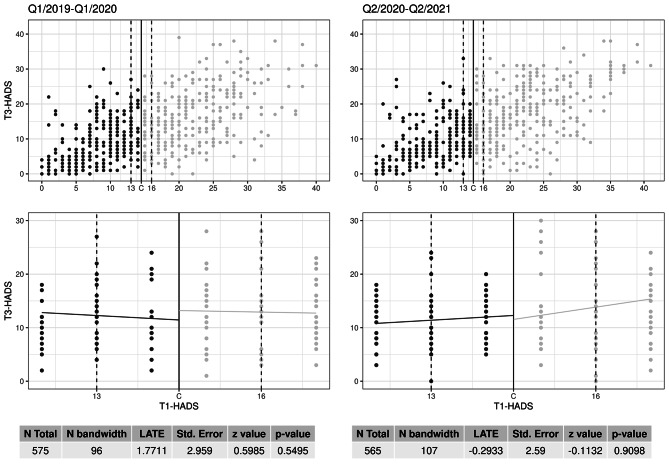
Scatter plot (four panels) of the primary outcome with the results of the regression discontinuity analyses (local average treatment effect - LATE) for the group before (left) and after (right) the pandemic for the patients’ baseline HADS scores (T1) and HADS scores after 12 months of treatment (T3) * Below Threshold (HADS ≤ 14): control (black); above threshold (HADS ≥ 15): intervention (grey)

Scatterplots with all cases of the two groups according to time within the pandemic are shown (Fig. 2). In both groups, i.e. in the control group below the threshold and in the treatment group above the threshold, the higher the T1-HADS score, the higher the T3-HADS score. The second row of Fig. 2 then shows the applied RDD analysis in the previously defined relevant section (bandwidth) of the entire collective. The area of interest was the bandwidth from 13 to 16 points on the HADS scale. The pre-pandemic group consisted in total of N = 575 patients (50.4%), of which N = 96 patients lie within the pre defined bandwidth. The post-pandemic group had a total N of 565 patients (49.6%) and N = 107 patients within the bandwidth. Both groups show a discontinuity of the regression lines in the area around the threshold. However, the local treatment effect (LATE) measured in each case is not significant. In addition, only the local treatment effect of the post-pandemic group points in the right direction (LATE=-0.2933), as it is negative and thus indicates an improvement (reduction) in the HADS values.

### Mean HADS over time

**Fig. 3:**
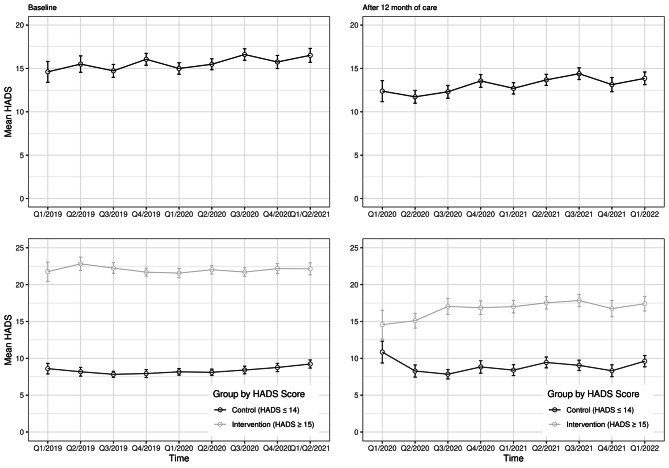
Mean Baseline and primary outcome HADS values by time. The two upper panels show the unstratified mean HADS scores at baseline (left) and after 12 months (right) over the course of the project. Below this is the stratified representation according to intervention and control group

In addition to the primary outcome, mean baseline and HADS scores after 12 months of treatment are compared in Fig. 3. Across all quarters, the overall mean HADS baseline scores are found to be in a very similar range between 15 and 16 points. 12 months after treatment, the HADS scores are also in a very similar range between 12 and 14 points. A direct comparison of the mean HADS scores at baseline and 12 months after treatment shows a reduction of about 2–3 points.

Comparing the control and intervention groups, the mean pre-treatment HADS scores in the intervention group at baseline (T1) are around 22 points across all quarters, well above the chosen threshold. The mean HADS scores of the control group, on the other hand, were around 8 to 9 points and thus, as with the intervention group, not in the range of 13–16 points relevant for RDD analysis. Comparing the mean HADS scores over time after 12 months of treatment between the two groups (intervention vs. control), it is visible that in the intervention group there was a reduction of the scores from over 20 points at the beginning of treatment to about 15 to 17 points after 12 months. In the control group, on the other hand, a slight deterioration, i.e. an increase in the scores, can be seen.

## Discussion

The nFC-isPO aimed to reduce anxiety and depression in patients with cancer as measured by HADS. However, due to the pandemic, it was suspected that patients would be exposed to additional psychological distress on top of the already high psychological distress caused by cancer, which could prejudice the treatment effect. Therefore, stratified analyses for primary outcome- (T3, 12 months of care) were performed in this article to examine the effects of COVID-19 in the isPO trial.

In contrast to the initial feedback from the four isPO care networks, the results of our descriptive analyses over time showed that the HADS scores in the post-pandemic group were only slightly higher on average or median overall than in the pre-pandemic group. This is also shown in other studies, such as Bargon et al.[17] being one of the few studies that examines the association between mental health in relation to cancer and the impact of the pandemic.

Many other studies investigated COVID-19 and the resulting deterioration in mental health [18, 19]. isPO, tried to reduce anxiety and depression in newly diagnosed patients with cancer with a stepped care approach. It was started before and carried on during the pandemic while facing additional challenges as a result of the pandemic. Even before the pandemic, supporting and counseling vulnerable groups, e.g. patients with cancer, was considered as very important [20]. In the face of additional sudden factors, like the pandemic, it is even more important to avoid additional stress[21, 22].

This is also reflected in the results of isPO, as the average anxiety and depression scores before the outbreak of the pandemic hardly changed compared to after. On the other hand, cancer studies without psycho-oncology stepped care approach, show significantly higher anxiety and depression scores due to an external factor such as COVID-19 [23]. It can be concluded that needs-oriented psycho-oncological support has a positive impact and reduces anxiety not only with regard to the cancer diagnosis, but also with regard to other medical factors/problems that have a negative impact on anxiety and depression, such as COVID-19 [24–26]. This implies that because the isPO-patients already received personalized psycho-oncological care during the pandemic, the HADS scores did not increase significantly compared to patients who received a cancer diagnosis during the pandemic without additional psycho-oncological care (no nFC-isPO) [27, 28].

Regarding the results of the RDD analysis, the comparison of the pandemic groups showed that the discontinuity, i.e. treatment effect, increased in the pre-pandemic intervention group instead of decreasing as expected. In comparison, the post-pandemic group showed the expected decrease in HADS scores in the intervention group. This outcome might be caused by the fact that the patients who had completed the HADS questionnaire at T1 pre-pandemic almost all only completed the T3 survey during, many also right at the beginning, of the pandemic. That is, the higher HADS scores could be due to additional stress from the pandemic. The group during the pandemic, on the other hand, filled out all questionnaires after the start of the pandemic, so that the pandemic situation was still tense, but no longer completely new. Moreover, by the time the pandemic started, nFC-isPO was already more advanced and matured within the four sites. Therefore, working routines saved the ongoing of the intervention with high quality [29].

### Limitations

A major limitation of the study was the high number of cases required for conducting RDD analysis. Originally, a case number of over 3,500 patients was planned in order to have sufficient cases for the RDD analyses (power calculation). After adjustments to the sample size due to poor enrollment, including the pandemic and the complexity of the nFC-isPO, only about half (N = 1,757) of the originally planned sample size could be enrolled. Although this was the minimum number of cases for the relevant RDD analyses, RDDs still benefits from the highest case numbers possible.

Another limitation for the proof of effectiveness was the patient group selected for ethical reasons. In the range between 13 and 16 points of the HADS, the treatment group showed a rather low need for psycho-oncological care. A more targeted examination of more severely stressed cases showed that the care offers were taken up and also showed corresponding effects (see summative study report [12]). Furthermore, the effectiveness of a psychotherapeutic intervention cannot be presented without the effect of time (in this case 12 months) and the effect of individual dose (number of therapeutical talks). Finally, the analyses conducted here cannot conclusively clarify whether the short-term reported increase in HADS scores by isPO-staff during pandemic reflects fear of additional illness and the impact on cancer treatment, or whether scores increased in the short term due to fears of poor accessibility to doctors or other cancer support services.

## Conclusion

Patients with cancer are considered as vulnerable group who suffer from anxiety and stress very suddenly [30], regardless of other external factors such as the COVID-19 pandemic presented here. Our results underline how reliable and resilient the nFC-isPO is to external influencing factors in everyday hospital practice. On the one hand, it turned out that HADS scores were not permanently higher, although this was perceived by the isPO care networks at the beginning of the pandemic. For another, despite the pandemic, the nFC-isPO led to a decrease in HADS scores on average over time. Interestingly, this was despite the need to switch from face-to-face care to sometimes exclusively telephone or video-based care [31, 32]. Therefore, the results underline the importance of early and personalized psycho-oncological support in order to reduce stress, anxiety and depression in patients with cancer.

## Data Availability

The datasets generated and/or analysed during the current study are not publicly available due to ethical and legal restrictions but are available from the corresponding author upon reasonable request.

## Abbreviations

CAPSYS^2020^: Computer-assisted assistance system in isPO
ISCED: International Standard Classification of Education
IQR: Interquartile range
isPO: Integrated, cross-sectoral psycho-oncology
HADS: Hospital Anxiety and Depression Scale
LATE: Local average treatment effect
nFC: New form of care
NRW: North Rhine-Westphalia
RDD: Regression discontinuity design
SD: Standard deviation
